# Implementation of a sepsis protocol in a community hospital using a Telemedicine Program

**DOI:** 10.1186/cc12210

**Published:** 2013-03-19

**Authors:** M Steinman, CA Abreu Filho, A Andrade, R Cal, N Akamine, J Teixeira, E Silva, A Kanamura, M Cenderoglo, C Lottenberg

**Affiliations:** 1 Hospital Israelita Albert Einstein, São Paulo, Brazil; 2 Hospital Municipal Dr. Moysés Deutsch, São Paulo, Brazil

## Introduction

Sepsis has high incidence around the world, approximately 400,000 new cases occur annually in Brazil. In developing countries it is still an important cause of death due to poor adherence to best practice medicine protocols, mainly in community hospitals. The Brazilian mortality rate of septic shock is around 60%. The aim of this study is to describe the first Brazilian initiative of implementation of a Telemedicine (TM) Project for therapeutic support of septic patients in a community hospital in São Paulo, Brazil.

## Methods

Since May 2012, a TM Program has been implemented at two hospitals in São Paulo - Hospital Municipal Dr. Moysés Deutsch (HMMD), a public, secondary hospital, and Hospital Israelita Albert Einstein (HIAE), a tertiary private philanthropic entity - due to a partnership with Brazilian Health Ministry. A TM Central Command was located at HIAE with Endpoint 97 MXP Cisco^® ^Solution and a mobile Intern MXP ISDN/IP Cisco^® ^for the remote hospital (HMMD) via dedicated GB/sec connection. Imaging examinations were evaluated using PACS technology. At HMMD, the Sepsis Protocol, based on the Surviving Sepsis Campaign, was started for every recruited patient admitted to the emergency department (ED) or the ICU, and assessed by the Central Command through TM with an experienced consultant.

## Results

Over a 6-month period, 33 patients with diagnosis of sepsis (including severe sepsis and septic shock) admitted to HMMD were evaluated by skilled doctors of HIAE via TM, and the Surviving Sepsis Campaign protocol was instituted. In total, 87.8% of the consultations originated from the ICU and 12.2% from the ED; 16 patients were male (48.4%), mean age was 44.2 years old. TM improved diagnosis in 12.1% and influenced clinical management in 87.9% of the cases; in 11 patients (33.3%) TM consultation led to antibiotic change. Hospital mortality rate was 33.3%.

## Conclusion

The implementation of the TM Project at a community hospital has a major impact in the eICU and eED Sepsis Management Program, and improved compliance with recommended care bundles.
